# Eine App für die informelle Pflege: Nutzungserfahrungen und -hindernisse aus Sicht von pflegenden Angehörigen

**DOI:** 10.1007/s00391-023-02249-1

**Published:** 2023-10-19

**Authors:** Annika Hudelmayer, Kenneth zur Kammer, Johanna Schütz

**Affiliations:** Bayerisches Zentrum Pflege Digital, Hochschule Kempten, Albert-Einstein-Str. 6, 87437 Kempten (Allgäu), Deutschland

**Keywords:** Häusliche Pflege, Pflegende, Digitale Technologien, Zufriedenheit, Qualitative Methoden, Home care, Caregivers, Digital technology, Satisfaction, Qualitative methods

## Abstract

**Hintergrund:**

Personen, die an informellen Pflegearrangements beteiligt sind, werden als Zielgruppe für digitale Angebote gesehen. Pflegende An- und Zugehörige übernehmen häufig die Organisation und Koordination der Pflege, was mit einem hohen Informationsbedarf verbunden ist.

**Ziel der Arbeit:**

Die Studie geht der Frage nach, inwiefern die Nutzung einer digitalen Anwendung – insbesondere die dort aufbereiteten, pflegerelevanten Informationen – von pflegenden Angehörigen als Unterstützung im Pflegealltag wahrgenommen werden.

**Material und Methoden:**

Eine marktreife Smartphone-App wird von 16 pflegenden Angehörigen über mehrere Monate genutzt. Mithilfe qualitativer Interviews wird untersucht, welche Funktionen in realen Pflegesituationen genutzt und als Mehrwert empfunden werden. Die Auswertung erfolgt mithilfe der Inhaltsanalyse nach Kuckartz.

**Ergebnisse:**

Die App wird punktuell genutzt, und die Zufriedenheit mit den darin aufbereiteten Informationen ist u. a. von der Pflegesituation abhängig. Ein Mehrwert und eine Entlastung durch die App werden v. a. zu Beginn einer Pflegesituation gesehen. Die Teilnehmenden weisen auf bisher nicht integrierte, aber wünschenswerte Anforderungen, z. B. regionale Informationen oder individuelle Anpassungen, hin.

**Diskussion:**

Damit digitale Unterstützungsmöglichkeiten ihr erhofftes Potenzial entfalten können, müssen diese den an informeller Pflege Beteiligten frühzeitig bekannt sein. Weitere Voraussetzungen dafür, dass Pflege-Apps von der Zielgruppe als Mehrwert wahrgenommen werden, sind eine vertrauenswürdige Darstellung der Inhalte, deren Individualisierbarkeit und Aktualität.

## Abstract (en)

**Background:**

A target group for digital services are people who are involved in informal care arrangements. Family caregivers often undertake the organization and coordination of care, which is associated with a high need for information.

**Objective:**

The study examined the extent to which family caregivers perceive the use of a mobile digital application as facilitating day to day care, in particular regarding information needs.

**Material and methods:**

A market-ready mobile smartphone app was used by 16 informal caregivers over several months. Qualitative interviews were conducted to investigate which app features are being used in real informal care situations and which are perceived as added value. The analysis was carried out with content analysis according to Kuckartz.

**Results:**

The app was sporadically used and satisfaction with the information it provides depends on the care situation itself. The participants see added value and facilitation through the app mainly at the beginning of a care situation. They expressed the wish for requirements that have not yet been integrated, e.g., regional information or individual adaptations.

**Conclusion:**

For digital support options to develop the desired potential, those involved in informal care must be aware of them at an early stage. Further prerequisites for care apps to be perceived as added value by the target group are a trustworthy presentation of the contents, the customizability and that they are up to date.

Große Hoffnungen werden in digitale Technologien im Bereich der Pflege älterer Menschen gesetzt. Neben der professionellen Pflege gelten auch Pflegebedürftige sowie pflegende An- und Zugehörige in häuslichen Pflegearrangements als Zielgruppe digitaler Unterstützungsangebote. Trotz wachsender Angebote und politischer Bestrebungen sind digitale Pflegeanwendungen bislang kaum verbreitet. Im Rahmen einer Interviewstudie wurde untersucht, inwiefern pflegende Angehörige von einer App hinsichtlich pflegebezogener Informationen profitieren und an welchen Stellen sich Hürden in der Nutzung ergeben.

## Hintergrund und Studienziel

Digitale Anwendungen sollen nicht nur in der Aufrechterhaltung einer selbstständigen Lebensführung in der eigenen Häuslichkeit unterstützen, sondern auch denjenigen Entlastung bringen, die Pflege leisten [7, 10]. Trotz heterogener Bedarfe und Lebenssituationen verdeutlichen der nationale und internationale Forschungsstand übereinstimmend, dass dennoch häufig ähnliche Herausforderungen in der informellen Pflege bestehen: Pflegebedürftige und Angehörige sind mit unübersichtlichen Angebotslandschaften zu Unterstützungsleistungen und fragmentierten Zuständigkeiten bei finanziellen, materiellen und Beratungsleistungen konfrontiert. Daraus resultieren ein hoher Informationsbedarf zu Pflege- und Krankheitsverläufen, Rechts- und Versicherungsfragen oder Entlastungsangeboten sowie ein hoher organisatorischer und zeitlicher Aufwand für die Koordination der Pflege [2, 4, 6, 14–17].

Zahlreiche Anbieter digitaler Angebote haben es sich zur Aufgabe gemacht, Lösungen für diese Informations- und Koordinationsbedarfe zu entwickeln [1, 18]. Auch politische Akteure setzen große Hoffnungen in digitale Anwendungen und fördern diese durch die Schaffung rechtlicher Rahmenbedingungen, etwa für digitale Pflegeanwendungen (DiPA). Hierbei handelt es sich um native Apps, Desktop- oder Browser-Anwendungen und zugehörige Hardware, die der Stabilisierung einer häuslichen Pflegesituation dienen sollen. Zielgruppen sind Pflegebedürftige und pflegende Angehörige bzw. weitere Beteiligte wie Ehrenamtliche oder professionelle Dienste [5]. Trotz dieser Hoffnungen in digitale Technologien sind sie in der häuslichen Pflege bisher wenig verbreitet [3, 11]. Dafür können verschiedene Gründe angeführt werden. Zum einen wird darauf hingewiesen, dass ein Großteil der in Forschungsprogrammen entwickelten Technologien nicht zur Marktreife gelangt, die Bedürfnisse der Zielgruppen unzureichend berücksichtigt [10, 18] und die Anwendungen unter verzerrten Bedingungen getestet werden [13]. Zum anderen erscheint es für informell Pflegende schwierig, verlässliche Informationen zu pflegebezogenen digitalen Technologien zu erhalten, sodass sie kaum zu deren Existenz und möglichen Nutzung sensibilisiert sind [10].

Vor diesem Hintergrund beschäftigt sich das Forschungsprojekt mit der Fragestellung, ob und inwiefern die Nutzung einer marktreifen digitalen Versorgungsanwendung von pflegenden Angehörigen als Unterstützung im realen Pflegealltag wahrgenommen wird, und welche Nutzungsbarrieren sich ergeben. Der Fokus des Beitrags liegt auf der Analyse der Informationsbeschaffung und der administrativen Tätigkeiten im Pflegealltag.

## Studiendesign und Methoden

Ausgangspunkt ist die Nutzung einer marktreifen, kostenfrei zugänglichen Smartphone-App durch pflegende Angehörige über einen Zeitraum von mehreren Monaten (Zeitraum: Oktober 2022 bis Mai 2023). Die App hat den Anspruch, verschiedene Bedürfnisse informell Pflegender in einer digitalen Anwendung zu vereinen, und umfasst folgende Funktionen: einen Ratgeber und Checklisten zu pflegerelevanten Informationen, einen Terminplaner, einen Chat und eine Dokumentationsfunktion zur Koordination der Pflege innerhalb eines privaten Netzwerks. Daneben können sich die App-User:innen in einem von Pflegefachkräften und technischem Support moderierten Forum austauschen und Fragen stellen. Der Informationsbedarf soll insbesondere gedeckt werden durch die Ratgeberfunktion, die kurze Texte zu Themen wie altersspezifische Erkrankungen, Leistungsbeantragung oder Gesundheitsförderung umfasst. Die Inhalte sind thematisch in Kapitel strukturiert, die aus kurzen Textblöcken bestehen. Sie erlauben einen grundlegenden Einblick in ein spezifisches Themenfeld (z. B. Umgang mit Demenz, Pflegehilfsmittel). Die Nutzenden haben die Möglichkeit, sich diese Texte über eine Vorlesefunktion anzuhören. Videos oder Abbildungen sind nicht enthalten. Die App ist im Kacheldesign gestaltet und geprägt durch zahlreiche bunte Illustrationen im Comic-Stil, die die Inhalte grafisch erkennbar machen. Die Inhalte der App werden regelmäßig durch neue Themen und Funktionen ergänzt.

Die Rekrutierung erfolgte im süddeutschen Raum über regionale Zeitungsaufrufe, soziale Medien, Flyer sowie Multiplikator:innen aus dem Gesundheits‑, Pflege- und Bildungsbereich. Insgesamt nahmen 18 pflegende Angehörige (17 Frauen, ein Mann) im Alter zwischen 45 und 65 Jahren teil; diese pflegten jeweils einen oder beide Elternteile. Die Versorgung fand überwiegend im gleichen Haushalt oder in der näheren Umgebung statt, wobei 3 Teilnehmende mehr als 1 h Fahrtzeit von der pflegebedürftigen Person entfernt wohnten. Die bisherige Pflegedauer betrug zwischen 4 und 5 Jahre. Um die Nutzung der App unter möglichst alltagsnahen Bedingungen untersuchen zu können, erhielten die Teilnehmenden keine Schulung oder Einführung in die Anwendung. Wie allen regulären User:innen standen ihnen eine kurze, in der App integrierte Beschreibung der Funktionen und der technische Support des Anbieters zur Verfügung. Teilnahmevoraussetzungen für die Studie waren der Besitz eines funktionsfähigen Smartphones oder Tablets sowie die Bereitschaft, die App über mehrere Monate im Alltag zu nutzen. Entsprechend kann von grundlegenden Digitalkompetenzen unter den Teilnehmenden ausgegangen werden.

Im ersten leitfadengestützten Interview wurden die Teilnehmenden vor der App-Nutzung zu ihrer aktuellen Pflegesituation, den damit verbundenen Herausforderungen und ihren bisherigen Erfahrungen mit Pflege-Apps befragt. Nach einer 3‑ bis 5‑monatigen Nutzungsphase wurde ein zweites Interview zu den Erfahrungen mit der App durchgeführt. Von 18 Teilnehmenden nahmen 16 an beiden Interviews teil. Die Interviews wurden transkribiert und mithilfe der inhaltlich strukturierenden qualitativen Inhaltsanalyse nach Kuckartz [12] ausgewertet.

## Ergebnisse

### Rolle von Informationen und administrativen Tätigkeiten im Pflegealltag

Die Befunde der ersten Interviewphase verdeutlichen, dass die gezielte Suche nach pflegerelevanten Informationen und die Ausführung administrativer Angelegenheiten Bestandteile des Alltags pflegender Angehöriger sind und eine zeitliche und belastende Herausforderung darstellen. Es wird berichtet, dass die Informationsbeschaffung „locker die Hälfte des eigentlichen Pflegens an der Person in Anspruch nimmt“ (T17_5). Während pflegende Angehörige bei praktischen pflegerischen Handlungen häufig Unterstützung erhalten (z. B. die Übernahme medizinischer Tätigkeiten durch professionelle Dienste), sind sie bei der Informationsbeschaffung und den administrativen Aufgaben weitgehend auf sich allein gestellt. Unter administrative Tätigkeiten fallen beispielsweise die Verwaltung von Versicherungen, Vereinbarung von Terminen, die Dokumentation des Gesundheitszustandes oder der Medikamenteneingabe sowie die Bezahlung von Rechnungen. Vor allem zu Beginn der Pflegeübernahme besteht ein hoher Informationsbedarf, der von großer Unsicherheit begleitet wird: „Also ich wusste überhaupt nicht, was es bedeutet, Pflegefall, was ein Pflegegrad ist …, man lernt da ja gar nichts drüber“ (T15_79). Die damit verbundene Belastung wird nicht nur durch Ausdrücke wie „Behördendschungel“ oder „Informationsdschungel“ (T16_35 und 43) deutlich, sondern spiegelt sich v. a. in folgender Aussage wider: „Diese Erkrankung, … dieser Verfall; … das ist alles in Ordnung. Was mich enorm anstrengt, ist dieses Behördliche“ (T17_53). Veränderungen im Gesundheitszustand der Pflegebedürftigen oder in den eigenen Lebensumständen machen die Informationsbeschaffung und die Erledigung behördlicher Angelegenheiten zu einem kontinuierlichen Prozess. Die Teilnehmenden äußern rückblickend, dass sie insbesondere zu Beginn der Pflege eine Hemmschwelle erlebten, sich bei Fragen oder Unsicherheiten an Fachpersonal zu wenden. Die Sorge bezog sich u. a. darauf, ‚falsche‘ oder unangemessene Fragen zu stellen und schambesetzte Probleme zu thematisieren.

### Informationsgewinnung vor der App-Nutzung

Die Interviews zeigen, dass das Internet nahezu durchgehend in unterschiedlichem Umfang zur Informationssuche genutzt wird. Recherchiert wird auf den Webseiten von überregionalen und kommunalen Gesundheitsinstitutionen (z. B. Krankenkasse, Gesundheitsministerium, Fachstelle), in Online-Ratgebern privater Anbieter und in sozialen Medien (z. B. Facebook-Gruppen, YouTube). Anlässe für Online-Recherchen sind häufig die Pflegegradbeantragung und die Suche nach Leistungen der Pflegekassen oder bestimmten Hilfsmitteln. Örtliche Beratungs- und Betreuungsangebote, ambulante Dienste oder Arztpraxen werden ebenfalls online identifiziert, die anschließende Kontaktaufnahme erfolgt jedoch überwiegend telefonisch oder persönlich. Auf regionale Angebote – beispielsweise Gesprächsgruppen oder Schulungsmöglichkeiten – werden Angehörige häufig auch über Printmedien (z. B. Broschüren, Flyer) aufmerksam.

Themenunabhängig berichten die Teilnehmenden, dass sie bei der Informationssuche an Grenzen stoßen, wenn sie konkrete oder individuelle Informationen benötigen oder Nachfragen haben. Die Recherche ist dann nicht nur zeitaufwendig, sondern häufig auch ergebnislos, wie eine Teilnehmerin erläutert: „Ich bin abendweise am Internet gehockt und habe geguckt und war dann wieder auf irgendwelchen falschen Seiten, wo es gar nicht gepasst hat“ (T16_37). In der Folge erfreuen sich Angebote, bei denen eine persönliche Kontaktaufnahme und direkte Rückfragen möglich sind, einer größeren Beliebtheit. Obwohl die Erreichbarkeit und lange Warteschleifen bei telefonischen Nachfragen als herausfordernd beschrieben werden, schätzen es die Interviewten als besonders wertvoll ein, wenn die Möglichkeit besteht, recherchierte Informationen auf die eigene Pflegesituation anzuwenden oder persönliche Herausforderungen zu besprechen. Dem Wissen von ausgebildeten Fachkräften verschiedener Disziplinen wird ebenso hohes Vertrauen entgegengebracht wie bestimmten Institutionen, beispielsweise Krankenkassen. Einen Großteil ihres pflegerelevanten Wissens erhalten die Angehörigen offenbar ‚nebenbei‘, wenn sie im Rahmen der Versorgung mit Fachkräften in Kontakt kommen.

Trotz der vielfältigen Informationswege war nur einer Teilnehmerin vor der Studie die Existenz von Apps zur Unterstützung der Pflege bekannt. Hinsichtlich der Sicherheit im Umgang mit Apps schätzen sich die Befragten im Vergleich mit ihrer Generation als weitgehend sicher ein. Im Vergleich zur jüngeren Generation sehen sie sich teilweise jedoch als weniger digitalaffin und geben an, dass für sie die Nutzung von Apps weniger intuitiv und mit größerem Aufwand verbunden sei. Nichtsdestotrotz berichten die Interviewpartner:innen über eine vielfältige App-Nutzung in ihrem Alltag.

### Erfahrungen mit der Pflege-App und Barrieren der Inanspruchnahme

Die Pflegenden bewerten die kurz und prägnant aufbereiteten Informationen in der App als übersichtlich und sehen insbesondere für Pflege-Neulinge den Vorteil, sich damit leichter im „Informationsdschungel“ (T16_43) zurechtzufinden. Die aufbereiteten Inhalte können dazu dienen, einen ersten Überblick über die Materie (z. B. Beantragung finanzieller Unterstützung, Pflegehilfsmittel) zu gewinnen. So resümiert eine Interviewteilnehmerin rückblickend: „Wenn ich die App von Anfang an gehabt hätte, hätte ich mir ziemlich viel Zeit gespart“ (T16_37). Die Informationstexte und Checklisten geben den Angehörigen zudem Begriffe und Erklärungen an die Hand, die eine (vertiefte) Auseinandersetzung mit verschiedenen Themen ermöglichen. Insbesondere im Hinblick auf die geäußerten Unsicherheitsgefühle könnte die App einen niedrigschwelligen und anonymen Einstieg bieten*: *„Du hast am Anfang einfach eine Hemmschwelle. …, da ist die App, wo du alles quasi anonym machen kannst und für dich rausholen kannst, sehr bequem“ (T7_69).

Angehörige, die sich seit Jahren beruflich und/oder privat mit Pflege beschäftigen, profitieren hingegen selten von den bereitgestellten Informationen. Sie sind bereits mit den Möglichkeiten der Beantragung finanzieller Leistungen oder möglicher Hilfsmittel vertraut und empfinden die aufbereiteten Inhalte als zu oberflächlich. Mit zunehmender Pflegedauer und einer komplexeren Versorgung steigt bei ihnen das Bedürfnis nach differenzierten Informationen sowohl zur medizinischen Versorgung als auch im Umgang mit spezifischen Erkrankungen. Während der mehrmonatigen Studiendauer gerät die App als mögliche Informationsquelle bei den Teilnehmenden in Vergessenheit, wird als überflüssig erlebt und als zeitraubender Mehraufwand empfunden. Aus Routine greifen die Pflegenden eher auf bereits gewohnte Informationskanäle zurück.

Das in die App integrierte moderierte Forum wird im Rahmen der Studie, aufgrund geringer Aktivitäten in diesem, selten zur Informationsbeschaffung genutzt. Die Angehörigen äußern zudem Bedenken, sich selbst aktiv im Forum einzubringen. Die Unsicherheiten beziehen sich sowohl auf die von ihnen bezweifelte Qualität der Antworten: „und dann meldet sich ja immer eine Manuela Moderatorin oder was? Weiß ja nicht, was die für Kompetenzen hat“ (T12_67) als auch die Ängste hinsichtlich der Reaktionen. Zudem besteht der Wunsch, die eigene Situation nicht nach außen zu tragen: „Ja, es ist sehr persönlich und … die müssen auch nicht alles wissen“ (T12_174). Insgesamt wurde eine langfristige Nutzung der App weitgehend abgelehnt.

### Wünsche für eine bedarfsgerechtere Entwicklung digitaler Unterstützung

Die Auswertung der Interviews identifizierte Wünsche der pflegenden Angehörigen für zukünftige (Weiter‑)Entwicklungen von Pflege-Apps. Dies betrifft die Darstellungsform und -tiefe der informationsbezogenen Inhalte. So wird mehrfach der Wunsch nach einer vertiefenden Ausarbeitung einzelner Themen geäußert. Darüber hinaus wird auf die Vorteile einer verstärkten Einbindung von Bildern und Videos, insbesondere zur Darstellung praktischer Tätigkeiten, hingewiesen. Speziell bei Pflegeerfahrenen besteht Bedarf nach Informationen zu individuellen Herausforderungen spezifischer Pflegesituationen und ‚Insider-Tipps‘, so z. B. „alltagspraktische Tipps, die nicht auch in jedem Buch stehen“ (T20_31). In diesem Zusammenhang wird von den Pflegenden u. a. der Wunsch geäußert, dass die innerhalb der App kurz gehaltenen Ratgebertexte auf weiterführende, qualitativ geprüfte Webseiten verweisen könnten. Eine kontinuierliche Aktualisierung der App-Inhalte, z. B. im Hinblick auf gesetzliche Neuerungen, wird zudem gewünscht. Dadurch könnte sich eine App, wie von den Teilnehmenden betont, gegenüber Printmedien und insbesondere Büchern auszeichnen. Eine mögliche Einbindung regionaler Informationen wird von den Teilnehmenden als Mehrwert angesehen und teilweise sogar als Voraussetzung für eine langfristige Nutzung betrachtet. Der Wunsch nach einer lokalen Übersicht zu Pflege- und Unterstützungsangeboten wird in nahezu allen Interviews angesprochen. Allerdings äußern die Befragten bereits im Vorfeld Zweifel daran, ob eine tagesaktuelle Darstellung verfügbarer Angebote realisierbar sei. So wird z. B. die Einbindung von Pflegediensten in die App aufgrund deren wahrgenommener Überlastung von vornherein als unrealistisch eingeschätzt.

## Diskussion

### Interpretation der Ergebnisse

Im Vergleich zu anderen Studien, in denen häufig die kurze und alltagsferne Nutzung kritisiert wird [13], bieten die hier dargelegten Studienergebnisse Einblicke, wie pflegende Angehörige eine App unter Alltagsbedingungen wahrnehmen, und auf welche Hürden und Barrieren sie stoßen.

Die Befunde verdeutlichen erneut den hohen Bedarf der Angehörigen nach zuverlässigen Informationen. Insbesondere zu Beginn der Pflege wissen die Angehörigen nicht, wo sie Informationen erhalten können, und auch die Vielzahl administrativer Tätigkeiten stellt sie vor große Herausforderungen.

Hinsichtlich des Informationsbedarfs, der durch eine App gedeckt werden soll, lassen sich zwei grundsätzliche Wünsche unterscheiden: allgemeine, regionsunspezifische Informationen, z. B. zur Beantragung eines Pflegegrads, und ortsbezogene Informationen. Während den allgemeinen Informationen in der App zum Pflegebeginn ein großes Potenzial zugeschrieben wird, möchten pflegeerfahrene Angehörige Fragen zur individuellen Anwendung gesetzlicher Regelungen an Fachkräfte unterschiedlicher Disziplinen richten. Zwar ist dies im Fall der hier genutzten App bereits in Ansätzen möglich, jedoch scheuen sich die Teilnehmenden, Fragen in einem, von der App bereitgestellten, öffentlichen Online-Forum zu stellen und haben Bedenken hinsichtlich der fachlichen Qualifikation der antwortenden Personen. Eine Kennzeichnung der Informationsquellen und der Qualifikation der dort agierenden Fachkräfte könnte das Vertrauen in die Inhalte erhöhen. Dies kann mit Blick auf das gesamte Angebotsspektrum deutschsprachiger Pflege-Apps (Stand Dezember 2018) unterstrichen werden. Eine Untersuchung hierzu zeigte, dass in nur 20 % der Apps evidenzbasierte Quellen transparent dargestellt werden [8].

Dass die Nutzung einer digitalen Anwendung insbesondere zu Pflegebeginn einen hohen Mehrwert mit sich bringt, zeigt sich auch mit Blick auf den Bedarf der Interviewpartner:innen, in der App niedrigschwellig anonym Informationen zur eigenen Pflegesituation zu erhalten. Die geäußerten Unsicherheiten, sich anfangs mit Fragen an Fachkräfte zu wenden und persönliche, teils schambesetzte Themen zu teilen [2], deuten auf Potenziale digitaler Anwendungen hin. Gleichzeitig werden aber auch Grenzen deutlich, da einige Teilnehmende die App bzw. bestimmte Funktionen explizit nicht nutzen möchten, um ihre Privatsphäre zu schützen. Dies betrifft insbesondere die Eingabe von Informationen zur Pflegesituation sowie explizit das Forum. Hier zeigen sich Unsicherheiten hinsichtlich der gefühlten Datensicherheit, die an verschiedenen Stellen in den Interviews deutlich werden. Die pflegenden Angehörigen zeigen sich demnach ambivalent hinsichtlich der empfundenen Vertrauenswürdigkeit, in diesem Rahmen persönliche Anliegen zu teilen. Auch der Wunsch nach regionalen Informationen innerhalb der App und die gleichzeitigen Bedenken, ob diesen Informationen im Falle ihrer Verfügbarkeit vertraut werden kann, weisen auf die vielschichtigen Herausforderungen hin, die mit den Hoffnungen in digitale Anwendungen verbunden sind.

So wird deutlich, dass die an der Studie teilnehmenden Angehörigen keinen unvoreingenommenen Blick auf digitale Pflegeanwendungen haben (können), sondern von ihren Pflegeerfahrungen geprägt sind. So etwa wie die von vornherein als unrealistisch eingeschätzte Einbindung von Pflegediensten in die App. Ebenso kristallisieren sich in diesem Zusammenhang Bedenken hinsichtlich der Verlässlichkeit digitaler Informationen und digitaler Informationsvermittlungsprozesse heraus. Das Vertrauen in analoge Informationswege wird u. a. mit jahrzehntelanger Erfahrung und einem damit verbundenen intuitiveren Handeln begründet, das – vor dem Hintergrund einer belastenden Pflegesituation – praktikabler erscheint. Generell spiegeln sich hier Vorerfahrungen der Befragten mit der Aktualität von Informationen im Internet und die langjährigen Erfahrungen der Beteiligten mit der fragmentierten Landschaft des deutschen Pflegesystems wider.

Hervorzuheben ist das von Einzelmotiven unabhängige Gesamtergebnis, dass kaum eine der teilnehmenden Personen die App tatsächlich und längerfristig für ihre häusliche Pflegesituation nutzen wollte, und dass es einige Hürden zu überwinden gilt, bevor digitale Anwendungen die in sie gesetzten Erwartungen erfüllen.

### Grenzen der Untersuchung

Die Studie weist Grenzen auf, die zugleich auf die Grenzen digitaler Pflegeanwendungen für die informelle Pflege hindeuten. Das Studiendesign war so angelegt, dass nur Personen mit internetfähigen Smartphones und ausreichenden digitalen Kompetenzen teilnehmen konnten. Ziel der Studie war es, die Teilnehmenden eine frei verfügbare App unter möglichst alltäglichen Bedingungen und ohne spezielle Unterstützung nutzen zu lassen. Dennoch ist zu bedenken, dass Personen ohne Internetzugang, ohne technische Ausstattung und Kompetenzen derzeit von digitalen Unterstützungsangeboten ausgeschlossen sind und sich damit digitale, soziale Ungleichheiten manifestieren [20].

Trotz intensiver Rekrutierungsbemühungen konnten keine älteren Pflegenden, d. h. Personen über 65 Jahre und keine Ehepartner:innen für die Teilnahme gewonnen werden, obwohl Ehefrauen – neben erwachsenen Kindern – die größte Gruppe der pflegenden Angehörigen darstellen [9].

Nichtsdestotrotz weisen die dargestellten qualitativen Befunde eine Vielzahl von Überschneidungen mit den Ergebnissen einer groß angelegten Mixed-methods-Studie auf, bei der mehr als 100 pflegende Angehörige und Pflegebedürftige die gleiche und eine weitere App für die familiale Pflege genutzt und bewertet haben [19].

## Fazit für die Praxis

- Den an der Studie teilnehmenden pflegenden Angehörigen waren Pflege-Apps vor der Studienteilnahme nicht oder kaum bekannt. Um die prognostizierten Potenziale digitaler Technologien zu entfalten, sollten diese Angebote für die häusliche Pflege stärker bekannt gemacht werden, damit sie bereits zu Beginn der Pflege als eine Unterstützungsoption zur Verfügung stehen.
- Eine Kennzeichnung der Informationsquellen und eine unabhängige Prüfung der Qualität digitaler Anwendungen könnten das Vertrauen in die Angebote und deren Inhalte erhöhen.
- Die Einbindung regionaler Pflegeangebote sowie eine Sicherstellung der stetigen Aktualisierung und Individualisierbarkeit von Informationen würden digitale Anwendungen stärker an den tatsächlichen Bedarfen der Nutzenden ausrichten.
- Nur eine allgemeine Reduzierung der Fragmentierung von Zuständigkeiten und Bürokratie in der häuslichen Pflege kann zu nutzer:innenfreundlichen digitalen Angeboten führen.
